# Hard SyDR: A Benchmarking Environment for Global Navigation Satellite System Algorithms †

**DOI:** 10.3390/s24020409

**Published:** 2024-01-09

**Authors:** Antoine Grenier, Jie Lei, Hans Jakob Damsgaard, Enrique S. Quintana-Ortí, Aleksandr Ometov, Elena Simona Lohan, Jari Nurmi

**Affiliations:** 1 Electrical Engineering Unit, Tampere University, 33720 Tampere, Finland; hans.damsgaard@tuni.fi (H.J.D.); aleksandr.ometov@tuni.fi (A.O.); elena-simona.lohan@tuni.fi (E.S.L.); jari.nurmi@tuni.fi (J.N.); 2 Parallel Architectures Group, Universitat Politècnica de València, 46010 Valencia, Spain; jlei@disca.upv.es (J.L.); quintana@disca.upv.es (E.S.Q.-O.)

**Keywords:** benchmarking, computational complexity, field-programmable gate array (FPGA), global navigation satellite system (GNSS), open-source software

## Abstract

A Global Navigation Satellite System (GNSS) is widely used today for both positioning and timing purposes. Many distinct receiver chips are available as Application-Specific Integrated Circuit (ASIC)s off-the-shelf, each tailored to the requirements of various applications. These chips deliver good performance and low energy consumption but offer customers little-to-no transparency about their internal features. This prevents modification, research in GNSS processing chain enhancement (e.g., application of Approximate Computing (AxC) techniques), and design space exploration to find the optimal receiver for a use case. In this paper, we review the GNSS processing chain using SyDR, our open-source GNSS Software-Defined Radio (SDR) designed for algorithm benchmarking, and highlight the limitations of a software-only environment. In return, we propose an evolution to our system, called Hard SyDR to become closer to the hardware layer and access new Key Performance Indicator (KPI)s, such as power/energy consumption and resource utilization. We use High-Level Synthesis (HLS) and the PYNQ platform to ease our development process and provide an overview of their advantages/limitations in our project. Finally, we evaluate the foreseen developments, including how this work can serve as the foundation for an exploration of AxC techniques in future low-power GNSS receivers.

## 1. Introduction

Nowadays, many kinds of electronics use satellite positioning systems or GNSSs for positioning and timing purposes [1]. Off-the-shelf GNSS receiver chips are available in a great variety for integration in embedded devices and for different applications, e.g., high-precision, autonomous driving, low-power, etc. The chips are implemented as Application-Specific Integrated Circuits (ASICs) for maximum energy efficiency but, as a result thereof, they allow neither inspection nor modification of their internal features. This is ideal from the point of view of an industry that wishes to protect its intellectual property but obstructs or hinders research, as it makes infeasible the exploration of the processing algorithm design space on this kind of platform.

Understanding the performance–energy trade-offs in GNSS receivers is becoming increasingly important as GNSS receivers are expected to be integrated into an ever-increasing number of battery-powered embedded devices [2]. Yet today, GNSS receivers remain some of the most energy-consuming sensors [3]. A GNSS receiver’s software and power consumption are influenced by several factors such as the user requirements, operating environment, and receiver electronics. Identifying the software configuration that maximizes performance and minimizes computational burden is complex and it may differ for each use case.

Software-Defined Radios (SDRs) are used extensively to test new Digital Signal Processing (DSP) algorithms in all fields of communication, including a GNSS. However, despite how several such tools are available in open-source [4,5,6,7,8], most of them suffer from at least one of these issues, as reviewed in [9]: lack of maintenance, little-to-no hardware integration, architecture different from an actual receiver, or not being designed for benchmarking. By benchmarking we refer to comparing the performance of various algorithms over a set of Key Performance Indicators (KPIs) of interest. Benchmarking is particularly interesting for our research goal of exploring the GNSS processing chain to find points where energy consumption could be reduced, for example, through approximation.

The interest in using approximation to increase the performance computing systems has grown with the decline in the historical trends in Moore’s law and Dennard scaling [10]. Nowadays, approximation, a term contained in the Approximate Computing (AxC) paradigm, refers to trading off small, constrained computational errors for reductions in the execution time, circuit area, or energy consumption of applications resilient to such errors [11]. While such techniques have been applied extensively to the Machine Learning (ML) and DSP domains [10], we notice a distinct lack of work using AxC in GNSS processing and highlight this as a promising direction for future work.

The goal of this paper is to provide additional details on our motivations for mixing software and hardware for benchmarking purposes in the field of GNSS processing and to document our conversion progress using High-Level Synthesis (HLS) and *PYNQ* [12] targeting an Field-Programmable Gate Array (FPGA), describing the barriers and ways around them that we have explored, the current architecture, and the initial results from its hardware implementation. We keep our prior focus on a partial conversion that enables collecting hardware-related KPIs of core algorithms implemented on FPGA while keeping benchmarking tasks in a rich and high-level environment. In summary, our contributions are as follows:
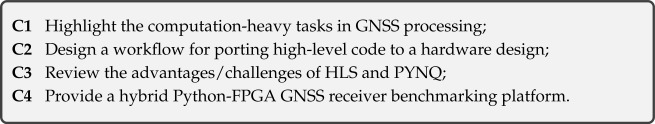


### 1.1. Related Work

For performance and efficiency reasons, most GNSS receivers are implemented in hardware rather than software. GNSS algorithm benchmarks can benefit from taking this into account as hardware implementations permit estimating additional KPIs and, thus, enhancing comparability. While it would be unreasonably time-consuming and costly to produce ASICs for this purpose alone, FPGAs provide a suitable alternative platform given their inherent low-effort reconfiguration characteristics.

The PYNQ workflow, designed by AMD (Santa Clara, CA, USA), for hardware–software co-design enables Python-based interaction with computationally heavy *kernels* executed on the embedded FPGA in AMD Zynq chips [12]. This workflow has rapidly grown popular in several research fields, most prominently in the ML domain whose algorithms conform well to its dataflow processing style. These algorithms can benefit significantly from the PYNQ workflow’s scalability and native support for custom low-precision number formats [13]. These features allow for speeding up exploration of the design space of convolutional neural networks [14] and increasing accelerator throughput with AxC techniques [15].

Following the recent launch of the RFSoC development platform [16], the PYNQ workflow is also increasingly being used in the communications domain for, e.g., accelerating an SDR’s Fast Fourier Transform (FFT) and signal modulation operations. This is illustrated by Goldsmith et al., whose open-source SDR permits software control of radio functionality and real-time radio signal visualizations [17]; Šiaučiulis et al., who utilize the FPGA to implement high-speed packet processing for networking [18]; and Chang and Chou, who propose a PYNQ-enabled mmWave-capable 5G transceiver [19]. These works utilize the PYNQ workflow to simplify the development of proof-of-concept systems with user-friendly interfaces. Our goals align well with this trend.

### 1.2. Prior Work

In prior work [9], we introduced the concept of our SDR named “SyDR”, standing for “SDR with Python”, and designed it for GNSS processing. We analyzed existing GNSS SDRs and the benefits of our architecture. The objectives of SyDR are (1) to be a receiver described in a high-level language, in our case Python 3; (2) to be open-source [20]; and (3) to serve as a playground for algorithm benchmarking. Being aware of the significant runtime overheads of Python [21], we already foresaw its conversion to a lower-level language (e.g., C or Rust) and its eventual implementation on hardware such as FPGAs.

We first addressed this conversion in [22] by outlining our workflow for porting the computation-heavy parts of SyDR into HLS-compatible C/C++ code [23], the targeted hardware architecture, and its integration into Python using PYNQ [24]. We motivated moving from software to hardware with three potential advantages, of which this paper retains two: (1) enable the use of AxC techniques only available in hardware [10,11] and (2) provide more realistic power and energy consumption estimates. Our focus on delivering a proof-of-concept solution has led us to disregard the third potential—processing acceleration—for now. We argue that this does not take away from the results presented herein, as neither absolute processing speed nor acceleration were the main aims of SyDR.

### 1.3. Paper Structure

The remainder of the paper is organized as follows. Section 2 gives background on the generic GNSS processing chain and receiver architecture implemented in SyDR and our motivation for moving it closer to hardware with the PYNQ environment. Section 3 describes our envisioned and currently implemented hardware architectures, while Section 4 details our conversion process. As the conversion is still ongoing and the architecture is yet to be completed and fully optimized, Section 5 only outlines the initial results. Section 6 discusses directions for future work, and Section 7 concludes the paper.

## 2. Background and Motivation

Similar to any modern radio system, GNSS receivers are composed of an analog stage, used for the recovery of Radio Frequency (RF) signals (e.g., antenna, amplifiers, Analog-to-Digital Converter (ADC)s), and a digital stage, used for DSP and extraction of the signal data. SyDR mainly focuses on the digital stage’s DSP algorithms. As detailed in our previous work [9], the goal of SyDR is not only to propose a software implementation of a GNSS receiver in a high-level programming language—Python—but also to render it modular enough to enable benchmarking of algorithms within a realistic GNSS receiver architecture. The benefits for researchers include, among others, avoiding redundant development and focusing research efforts on the underlying algorithms. Using a well-defined framework also ensures any changes are isolated to the algorithms, thus enhancing their comparability. We start by recalling the typical GNSS processing stages before diving into the motivations to go closer to the hardware through the PYNQ platform.

### 2.1. GNSS Processing Stages and Computational Complexity

**Figure 1:**
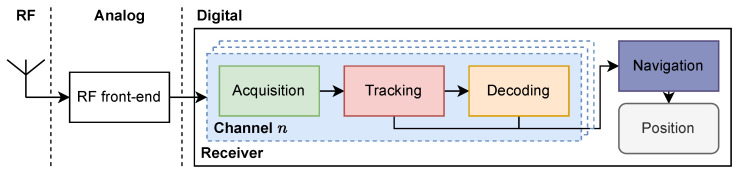
Typical GNSS processing chain, as implemented in SyDR.

A processing chain embedded in GNSS receiver can be simplified into the following four main processing stages, which are summarized in Figure 1 and as follows [5]:

1. **Acquisition:** Any new signal scheduled for tracking must first pass through an acquisition stage, which identifies the visible satellites in the sky and searches for the initial Doppler and code shifts of each of the satellites visible in the sky at the location of interest. The size of the acquisition search space depends on the current status of the GNSS receiver (i.e., cold/warm/hot start), and of the possible access to Assisted-GNSS data (e.g., cellular radio, internet). If this stage fails due to the signal not being present or being suppressed by noise, the receiver will not proceed to the next stage.
2. **Tracking:** Once the coarse parameters for the Doppler and code shifts have been found, their values must be fine-tuned and continuously tracked to precisely align the signal replica with the incoming signal to retrieve the navigation message (i.e., data bits). If the tracking is lost, the receiver reverts to the acquisition stage, though a very limited search space is needed in most cases. Otherwise, the receiver remains in this stage until shutdown.
3. **Decoding:** Recovery of the navigation data bits becomes possible when the replica perfectly aligns with the incoming signal. These bits encode the timestamp necessary to compute the measurements used for user positioning.
4. **Positioning:** Using the tracking measurements and the decoded navigation data from multiple satellites, the receiver can recompose the *pseudoranges* and compute a position (e.g., Standard Point Positioning (SPP), Differential GNSS (DGNSS), Precise Point Positioning (PPP)).

Steps 1, 2, and 3 should be performed concurrently for tracking each signal. In this paper, we encapsulate these functions within the concept of *channels*, where each channel is responsible for tracking the signal from one satellite.

The acquisition is often highlighted as the most computationally complex part [1] as it might involve a significant number of arithmetic operations for performing convolutions. While the use of the FFT in modern receivers dramatically reduces this complexity, from $O(n^{2})$ (Serial Search) to O(nlogn) (Parallel Code Phase Search (PCPS)) [1], the acquisition stage still requires more operations than the tracking stage. Moreover, using the FFT in hardware has proven challenging, as discovered in our experiments reviewed in Section 5. As tracking “only” requires a few correlation operations, its computational complexity will in any case be lower than the one in the acquisition stage. The actual complexities of the DSP operations of a GNSS receiver are highly dependent on the scenario assumptions (e.g., algorithms used, environment, Assisted-GNSS, user dynamics, etc.) [1].

Nevertheless, using the SyDR platform, it is possible to list these assumptions and have a better idea of the actual complexity in processing time. Absolute processing times are irrelevant, as they are affected by many factors (e.g., hardware technology, operating system, programming language). However, relative processing times can enable comparison between different algorithms of the same processing stage or between processing stages. The former allows for identifying an optimal algorithm based on predefined KPIs (e.g., complexity, quality, reliability); the latter helps us expose critical points of the processing chain with high computational complexity. Optimizing these points can lead to large energy savings with minimal negative impact on the results.

**Figure 2:**
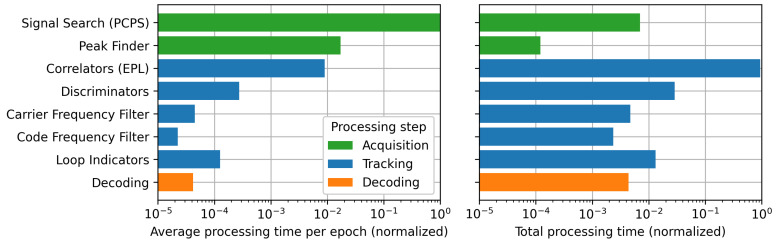
Processing time results from SyDR (Python) for each processing stage inside a channel: average processing time (**left**) and total processing time (**right**).

In Figure 2, we review the processing time needed for the different functions of SyDR. The processing is based on real data recorded in our lab and replayed in post-processing within SyDR. The details of the RF signal, algorithms, and parameters used are provided in Table 1. Execution time is measured using the time.perf_counter_ns functionality in Python, which is recommended in the Python PEP guidelines for performance comparison [25].

In these plots, we separate the processing stages into a number of functions within SyDR to achieve a more fine-grained resolution of the processing costs in and between the stages. We evaluate this processing cost over a scenario with 15 s of RF data (details in Table 1). We also propose two metrics to evaluate the processing cost: the average processing time per epoch (left) normalized by the highest-cost process (i.e., Signal Search), and the total processing time (right) normalized by the total processing time. An “epoch” is defined here as each millisecond of RF data fed to the channel. Normalization of the results is performed to ease comparison. We do not include the positioning stage in our comparison, as it is performed outside the processing flow of a single channel. However, we assume it is much less computationally expensive than both the acquisition and tracking stages, knowing the low rate with which the positioning is executed (∼1–10 Hz) compared with the other stages (∼1000 Hz) and the few arithmetic operations required.

While falling victim to the aforementioned limitations and pitfalls of comparing processing times of software, the results presented can still provide some gross/coarse insights on the promising regions for optimization. For instance, the assumption that acquisition is more costly than tracking is true and clearly visible in the left graph. In fact, the difference is greater than two orders of magnitude when compared to correlator operations. However, when integrating this processing time over several seconds of data, tracking has the highest total processing time by far. This is expected as the acquisition is performed only once at the beginning of this scenario, and the channel spends most of the processing time in the tracking stage. Yet, the important conclusion is that while acquisition might be more power-hungry than tracking, minor optimizations in the tracking stage could ultimately lead to considerably larger energy savings. Thus, while research over the last years has focused on reducing acquisition complexity [1], greater energy savings could be achieved with improvements in the correlator complexity.

Notably, several assumptions need to be taken into account for an enlightened interpretation of these results, besides the parameters listed in Table 1, such as the following:

- We use a simple PCPS algorithm for acquisition, to which there are many lower-complexity alternatives, such as QuickSynch [26]. Nevertheless, we minimize the complexity of the PCPS algorithm by performing no coherent/non-coherent integration and using only 1 ms of RF data. While this signal was chosen to have a high Signal to Noise Ratio (SNR), signals with lower SNRs would require increasing the integration time, which would lead to a significant increase in total processing time (right plot).
- We employ a complete scenario time of 15 s (i.e., 15,000 ms). This time was chosen empirically, as a bare minimum of 12 s is needed to confirm the first navigation message page in GPS L1 C/A signals [5] and, thus, the first Time of Week (TOW).
- Extending the scenario time to 60 s (or beyond) would drastically increase the total processing time spent in the correlators, rendering the acquisition stage relatively much less significant (right plot).

From this discussion, it is clear that SyDR can help assess the cost of each stage in the GNSS processing chain. However, given the high abstraction level and associated overheads of Python, benchmarking in SyDR is restricted to the software level. To propel our benchmarks to assess energy consumption better, we propose moving the processing to a more constrained environment, namely, a dedicated hardware platform. We denote this version of our tool “*Hard SyDR*”.

### 2.2. Moving Closer to Hardware

Thanks to the improvements in computers and microelectronics over the last 25 years, algorithm developments in GNSS processing have slowly transitioned from hardware to software implementation [9]. While this has greatly simplified the development and verification of new algorithms, it has complicated realistically estimating the cost of using a specific algorithm within the processing chain. Relative comparisons between algorithms can still be achieved, assuming they are developed with a similar quality (e.g., optimized resource usage) and benchmarked over identical datasets. However, absolute estimates of their energy consumption are not feasible, given the potential interference of concurrent operations running on the same processor.

Consequently, we consider moving the algorithms back to hardware for more realistic estimates of their costs. Indeed, doing so not only permits the collection of additional KPIs related to resource consumption and timing, but it can also help pinpoint any potential challenges pertaining to the hardware implementation of an algorithm not immediately visible from the software perspective. While one may argue that these metrics are better collected from the type of platform that the algorithms are expected to be implemented in, i.e., ASICs, for ease of use, we choose FPGAs as the target hardware platform. We acknowledge that, owing to their reconfigurability, FPGAs are known to be slower, and less area- and energy-efficient than ASICs. Yet, the differences are well-established in the literature [27]. Hardware simulations of ASICs and FPGAs are feasible but unattractive due to long execution time and occasionally doubtful accuracy [28,29].

Using FPGAs for GNSS receiver development is not new and has been performed in several SDRs. We have summarized examples of such in a previous work [9]. However, all previous implementations focus on real-time processing and, therefore, use FPGAs for hardware acceleration (e.g., GNSS-SDR [6], Tiira [30]). We take a different approach and propose to review the applicability of reconfigurable platforms like FPGAs for power estimation and benchmarking.

In the context of benchmarking, real-time processing is not a necessity, as it is more interesting to replay and post-process well-defined datasets to accurately compare algorithms. While post-processing can be carried out by a real-time processing system (i.e., using a Universal Software Radio Peripheral (USRP) to replay an RF signal), this approach over-complicates the software and overall system greatly with no added value to our analyses. This is the prime reason for developing SyDR instead of using a more advanced open-source tool, such as GNSS-SDR. However, our approach is not to be confused with GNSS-SDR [6], which used GNU-Radio to further replicate the behavior of a USRP using Python code. In our applications, we only target evaluating the energy efficiency of GNSS algorithms at the hardware layer and do not review any aspect of the hardware used for recording.

Interoperability and usability have been major drivers for the development of SyDR from the start, as defined in [9]. Consequently, with Hard SyDR, we look into a way to move from software to hardware while retaining modularity. The present work is a proof-of-concept that probes the feasibility of such developments.

### 2.3. The PYNQ Flow

To the best of our knowledge, no straightforward way to transform Python code into hardware designs exists. We are aware of several Python-embedded Hardware Description Languages (HDLs), including Amaranth [31], MyHDL [32], and Mamba [33], but their use in SyDR would require vast re-development efforts. Moreover, as we only aim for a partial conversion of SyDR, we need a software framework and a hardware platform that support low-overhead communication between each other and with which a synthesizable hardware design can be attained with minimal re-development efforts.

These characteristics match exactly the purpose of recent MPSoC and RFSoC systems proposed by the leading FPGA manufacturers, Intel and AMD, which integrate Central Processing Units (CPUs) with an FPGA on a single chip. These platforms enable previously unseen degrees of freedom for implementing processing in the CPUs and FPGA concurrently. For our purposes, the AMD Zynq platform is a relevant example of such a platform as it is easily accessible through the associated Python-embedded PYNQ flow [12,34]. Thus, we chose the AMD KV260 development board [35] to perform the first implementation of our system. This board is used as our target platform throughout the paper.

Zynq chips are logically split into two parts: the Processing System (PS) comprising general-purpose (ARM) CPU cores, caches, I/O devices, etc., and the Programmable Logic (PL) comprising an FPGA [34]. An Advanced eXtensible Interface (AXI)-based Network on Chip (NoC) serves to interconnect the PS’s devices with the PL. The PS–PL interface in particular comprises a total of nine master or subordinate interfaces with bit-widths of either 32 or 64 available from within the PYNQ environment [36], which is specified in more detail in [37], Chapter 35. The PYNQ flow abstracts reasoning about data movement over these interfaces as well as the dynamic loading of different designs (denoted by *overlays*) in the PL from Python code running in the PS [12]. These features render PYNQ an ideal fit for our proof-of-concept implementation and directly enable our partial conversion of SyDR with only the processing algorithms ported to an FPGA.

**Figure 3:**
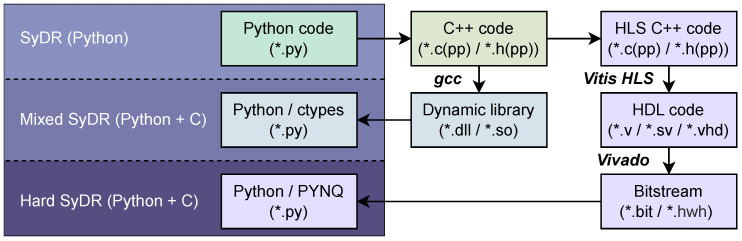
Conversion workflow for SyDR from pure Python to partial hardware implementation enabled by PYNQ and HLS.

However, to be implemented on an FPGA, a hardware design must be *synthesized* into a *bitstream* using AMD Vivado (version 2022.2). This requires algorithms to be written in an HDL like VHDL or (System)Verilog. This implies a step of design conversion either directly from Python to HDL, or from Python via a lower-level language to HDL. Although the first option is more direct, it generally involves long development cycles for design and verification. Instead, we opt for the second option and choose C++ as the intermediate language, as it permits us to shortcut the hardware design process by using HLS to generate HDL code [38]. Both conversion strategies are illustrated in Figure 3.

Using C++ and HLS has multiple benefits: (1) it reduces the required development efforts; (2) it enables using the AMD Vitis HLS (version 2022.2) flow to automatically configure the interfaces needed between the PS and the PL [12]; (3) it permits testing algorithms without an FPGA board using a library for interfacing external functions with Python, e.g., ctypes [39]; and (4) in the future, it may allow us to explore compiler-enabled AxC techniques such as precision tuning [40]. Moreover, HLS simplifies design space exploration into dataflows, pipelining, and initiation intervals of functions and loops with precompiler annotations [38]. This renders it an ideal fit for our proof-of-concept, benchmarking-focused use case.

We are aware that the typical HLS flow tends to produce hardware designs with lower performance and higher resource consumption than expert-written HDL equivalents [41]. Yet, as the latter would require intricate knowledge both of GNSS processing and development in HDLs, we see HLS as a reasonable shortcut to obtain coarse, yet indicative, hardware results. More importantly, we note that the HLS step *can* be circumvented by manual development, assuming the handwritten design remains compatible with the defined PYNQ interface.

## 3. Hard SyDR

The *Hard SyDR* [24] project is based on the *SyDR* [20] and *C SyDR* [23] projects (outlined in Figure 3), sharing their goals of implementing a modular SDR for benchmarking GNSS algorithms. While trying to minimize redundant developments, our goal is to mix programming languages to have the best of both worlds. For instance, while Python is known to have high overheads stemming from being interpreted rather than compiled and executed [21], its versatility and native support for numerous open-source libraries make it attractive for the first stages of algorithm development and file I/O and analytics (e.g., databases, plotting). Achieving similar functionality in lower-level languages like C/C++, or at the hardware level, is much more challenging.

With this in mind, we retain a large part of the tool in Python to deal with most of its user, file, and analysis features and port only the functions relevant for benchmarking outside of Python. As outlined in Section 2.1, these functions are encapsulated within channels. Yet, while C SyDR retains the control of these structures in Python, interfacing compiled C/C++ code with ctypes, this style of processing would require too many PS-to-PL data transfers in Hard SyDR. Instead, we port this control to the FPGA and enclose it in a new “*channel manager*” module, which we describe in more detail in the following.

### 3.1. Envisioned Architecture

**Figure 4:**
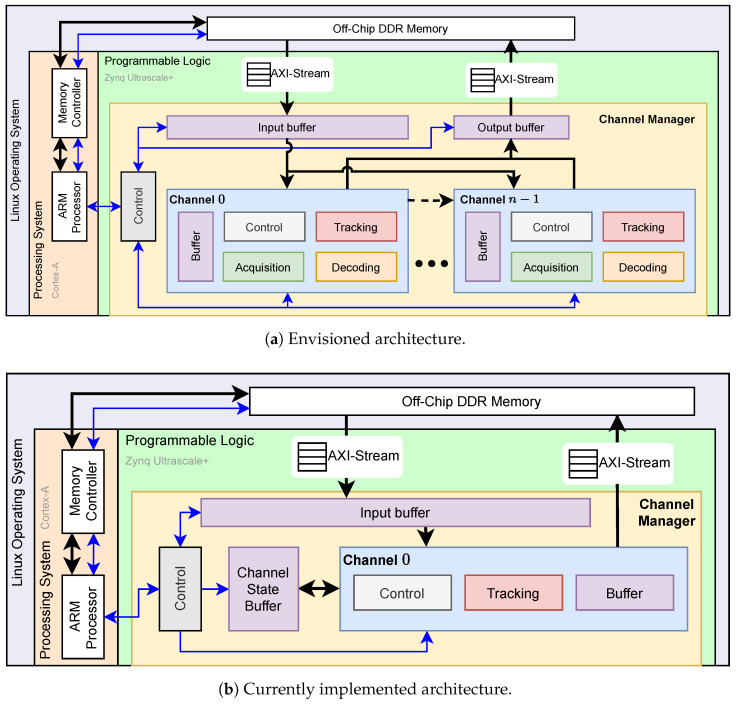
Overview of the hardware architecture for Hard SyDR accelerated on the Zynq platform.

With Hard SyDR, we aim for an architecture resembling that shown in the PL section of Figure 4a. We model this architecture after the structure of C SyDR but introduce several changes, especially to the top-level interfaces: where C SyDR itself can read and write data files, Hard SyDR requires a PYNQ driver to handle this. It is possible to allocate buffers in main memory and pass around pointers to these for the PL to access, but interfacing the PL through Direct Memory Access (DMA)-based AXI-stream transfers is usually more efficient, especially for large, contiguous chunks of data like the RF signals used in SyDR. As such, the aforementioned channel manager uses two such stream interfaces for input and output streams. Additionally, the channel manager hosts an input buffer, which is sized to match the data needs of the acquisition stage’s coherent/non-coherent integration range, and an output buffer, which stores just one response from each channel to be returned once their processing is finished.

The channels shown are independent but identical, i.e., they track different satellites starting from the same point in the RF signal till they have acquired a signal and its offset. This renders them highly suitable for operator or function reuse to avoid unnecessary duplication, but may prevent using a single input buffer due to expectedly irregular memory accesses. The former implies a potential for design space exploration to identify the best area-latency trade-off—or receiver architecture—taking the channels’ concurrency into account. For example, given its infrequent use, one acquisition stage may be sufficient for a handful of channels. The latter may require some degree of buffer duplication or, at the very least, additional control logic.

While our initial efforts target an architecture that solely responds to incoming RF data with channel configurations given at time of design-time, we envision being able to control its functionality at run-time using a third AXI-based interface. With the relevant control registers memory-mapped and accessible over this interface, a PYNQ driver can directly write values to them. Alternatively, the interface may transfer instruction-like messages to a top-level control unit responsible for distributing them to the channels.

Our design implements a finite-state machine behavior, unlike accelerators such as [15]. That is, the design must be able to receive Radio Frequency (RF) data a little at a time and only occasionally, when enough data have been provided, start processing and return one collective response. This differs from the streaming dataflow-oriented design style typically used in HLS flows but may be achieved with properly written code. Moreover, while using and implementing floating-point arithmetic on FPGAs is possible, its resource utilization is usually unreasonably high. As such, we plan to eventually abandon these types in favor of fixed-point alternatives available in AMD’s arbitrary precision libraries [38].

### 3.2. Current Architecture

The architecture currently implemented, shown in Figure 4b, significantly differs from the envisioned architecture. These differences, however, are primarily introduced deliberately to arrive at a minimally viable, intermediate design that nevertheless proves the practicality of our platform concept faster. First, the resulting design integrates only a single channel. This prevents parallel processing of multiple channels and positioning results, but it is sufficient for benchmarking intra-channel algorithms, such as the DSP-heavy acquisition and tracking stages. Second, the design does not implement a decoding block as we consider this less important in our current development process.

Third, and most importantly, the design does not integrate the acquisition module originally envisioned to be present in all channels, as shown in Figure 4a. This is mainly due to (1) difficulties related to hardware implementation of the FFT, namely its memory resource utilization, which would prevent simultaneous implementation of multiple channels, and (2) high noise levels resulting from zero-padding, which affect the acquisition performance, as will be detailed in Section 5. We consider two strategies to resolve this issue: (1) performing benchmarking of the acquisition and tracking operations separately by loading/unloading hardware layouts through the PYNQ flow during processing and (2) having a limited number of acquisition blocks that multiple channels share. Of these strategies, the second appears most attractive as the first option prevents easy re-acquisition if a channel’s tracking fails.

## 4. Development Details

We implement the architecture described above in C++ as a slight adaptation of C SyDR that takes the constraints of the FPGA and the limitations of the HLS flow into account. Subsequently, we use Vitis HLS to compile and convert the C++ code into an equivalent HDL description. This section covers the insights, challenges, and proposed solutions that our experiments with this conversion process to C/C++ and on to HDL have brought about.

### 4.1. Preparing Algorithms for HLS

The first step in our conversion involves translating Python code into C++. While this step implies a certain amount of redundant work, we found it rather straightforward given that the pure Python version of SyDR may be used as a golden reference when creating tests. Moreover, the mixed integration of Python with compiled C/C++ code works well for debugging as it limits the scope of changes to the individual functions of interest, while exploiting the Python environment for I/O and user interactions.

**Figure 5:**
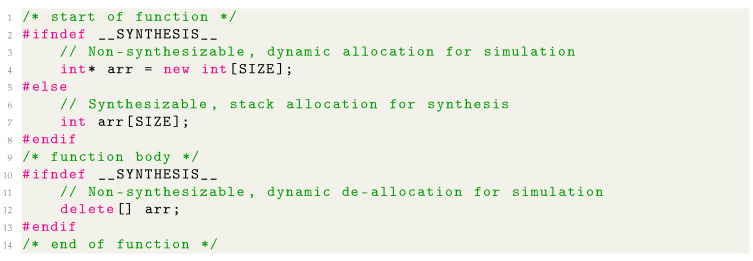
Example of using dynamic memory allocation for simulation only with the __SYNTHESIS__ macro.

Nevertheless, it is useful to keep the constraints of the HLS flow in mind already at this stage to avoid unnecessary re-development later on. These constraints involve several key concepts that are common and valid in software development but have no obvious equivalents in hardware; for example, recursion, unbounded loops, and dynamic memory allocation [38]. For C/C++, the former two are relatively easy to avoid while the latter implies eliminating functions such as ‘malloc/free’ or ‘new/delete’ and declaring all arrays, which typically map to some kind of memory, with sizes known at compile-time. A side effect thereof is that if the code is compiled and executed, the arrays will be allocated on the stack, whose permissible size is usually small. In Vitis HLS, this issue is most easily tackled by using preprocessor directives and the __SYNTHESIS__ macro that is defined only during synthesis to enable dynamic allocation during simulation [38]. Figure 5 illustrates this for the allocation using new and deallocation using delete[] of an integer array arr with SIZE elements.

The most challenging conversion step involves the FFT in the PCPS acquisition algorithm. While Python includes a well-established, flexible FFT algorithm in numpy, there is no comparably well-documented algorithm for C++. To match the Python implementation, we use the open-source PocketFFT [42], which numpy’s algorithm is based on [43], in C SyDR. However, as this algorithm relies on dynamic memory allocation, it is incompatible with the HLS flow. For this reason, we choose to replace it with AMD’s synthesizable FFT Internet Protocol (IP) block [44] in Hard SyDR; there will be more details on this later.

### 4.2. Synthesis of C/C++

After implementing these changes to the code, we turned our attention to Vitis HLS for synthesis. This led us to discover several additional restrictions of the tool: the hls_math header, which is meant as a synthesizable drop-in replacement of cmath, does not provide a full set of functions for complex data types [38]. Thankfully, for Hard SyDR this merely meant replacing two complex exponential functions with their equivalent real-valued cosine and sine functions. We also found that the compiler erroneously flagged a pointer reassignment as an unsupported pointer-to-pointer operation. Nevertheless, as this operation is poor code style even from a software development perspective, we have reworked the affected C SyDR code to avoid it too.

With the final changes in place, we implement the top-level channel manager function with the aforementioned streaming interfaces. For improved efficiency in data transfers to the PL, we pack eight 8-bit RF samples into one 64-bit word and unpack them in the channel manager. We also adapt the C SyDR main function into a testbench capable of interfacing the channel manager, passing its data from an RF data file and writing any returned values to another file. The testbench is used to verify the design either by executing it in software or by simulating its post-synthesis hardware implementation.

Next, we launch the synthesis flow itself, which, in addition to a set of HDL files, produces a useful report that includes the latency and estimated resource utilization KPIs for various functions and loop kernels. In accordance with the literature, however, we found that these metrics may be inaccurate [45]. Nevertheless, they indicate code regions with high potential for optimization either by manual revision or using HLS directives [38]. For use in an FPGA, the synthesized design must be packaged into an IP core. This standalone package contains the HDL version of the design and can be imported into AMD Vivado.

### 4.3. FPGA Design and Integration with PYNQ

**Figure 6:**
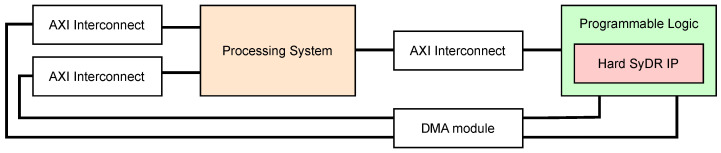
Block diagram of Hard SyDR for implementation in Vivado IP Integrator.

To target the Zynq platform, we use the Vivado IP Integrator tool to create a block design, consisting of our generated core, the PS, a DMA, and any AXI interconnect blocks necessary to establish communication. The resulting diagram is shown in Figure 6. The Python code running in the PS is in charge of instructing the DMA to perform streaming transfers of contiguously allocated chunks of main memory to the PL. The AXI interconnects handle transforming these transfers to ensure their compatibility with, e.g., the data widths of the PS and PL interfaces. Specifically, the Hard SyDR design occupies three of the PS’s high-performance AXI interfaces: one master interface (“M_AXI_HPM0_FPD”), for data and control transfers via the DMA to the PL, and two subordinate interfaces (“S_AXI_HP0_FPD” and “S_AXI_HP0_FPD”) for return data transfers, also via the DMA. Both data interfaces are manually specified to be 64 bits wide, while the control channel is automatically sized by Vitis HLS and Vivado.

Once assembled, the block diagram can be synthesized and implemented to target the desired device. The results include a bitstream and a so-called hardware header/handoff file, which includes information about the IP blocks and addresses of the memory-mapped registers included in the design. The PYNQ flow needs both these files to generate a driver to interface the design automatically. While we aim to incorporate this driver into SyDR, we use the Jupyter Notebook environment for initial testing as it lets us interact with the FPGA through a browser.

**Figure 7:**
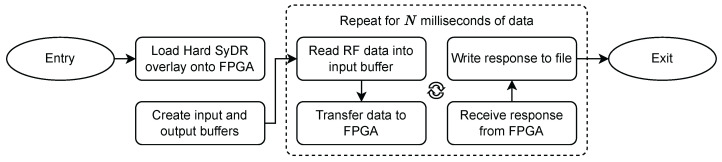
Simplified flow chart of the Jupyter notebook used to drive the Hard SyDR design.

Figure 7 shows a simplified flow chart of the Jupyter Notebook. The main elements of its code are included in Appendix A. We first program the FPGA with our bitstream and allocate arrays for inputs and outputs. Then, we loop over the processing steps for several milliseconds of RF data, writing the tracking results to a file.

## 5. Results of the Current Hard SyDR Implementation

In this section, we review the initial results computed using the current implementation of Hard SyDR. For the results presented in both sections, the GNSS algorithms assume the same parameters defined in Table 1.

### 5.1. Results of the Acquisition Stage

**Figure 8:**
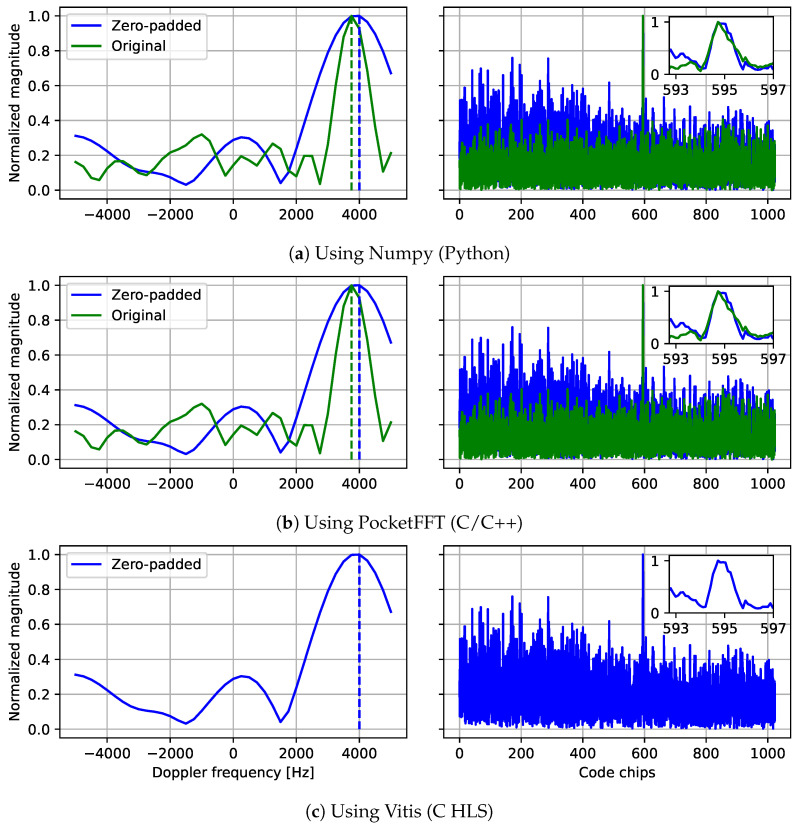
Comparison of acquisition results from different FFT implementations with and without zero-padding. Vertical dash lines represents the value found by the acquisition.

As detailed in Section 4, we encountered significant challenges in porting the acquisition step to the FPGA, which forced us to use AMD’s FFT IP block in the PCPS algorithm. However, this version of the FFT constrains its input signals to have a length equal to a power of 2 [44], as it implements the famous *Cooley–Tukey* algorithm [46]. This implies a need for padding shorter signals, often accomplished with zeros, to the next power of 2 [47]. In our case, using a 10 MHz sampling rate leads to 10,000 samples per millisecond of data. As the next power of 2 is 16,384, these samples must be padded with 6384 zeros to obtain a sufficiently long signal. In our implementation, these zeros are appended to the end of the original signal. We present results of the three different FFT implementations mentioned so far in Figure 8: the numpy FFT, the PocketFFT, and the AMD FFT IP, with and without zero-padding. These results were obtained using the Python SyDR implementation and simulations in the Vitis HLS software. We configure the AMD FFT IP block to use floating-point data and the following parameters: ordering_opt=natural_order, rounding_opt=convergent_rounding, arch_opt=radix_2_burst_io, has_nfft=true, and phase_factor_width=24. The notion of a code “chip” in this case is equivalent to the length of one bit in the Pseudo-Random Noise (PRN) code.

The results show correct correlation results in both code phase and Doppler estimation. However, zero-padding clearly leads to significantly wider peaks in the frequency domain, which can be explained by the substantial number of zeros needed to reach the 16,384 samples, i.e., more than 60% of the original signal length. An evident solution for these effects is to either raise the sampling rate closer to the next power of 2, or to reduce it below a previous power of 2. For example, using a sampling rate of 8 MHz instead of 10 MHz leads to only 8000 samples, less than 8192, per millisecond of data. Nevertheless, the results shown in all three implementations are consistent, thus validating our conversion.

Unfortunately, the AMD FFT IP block turned out to be challenging not only because of its input size constraints; concretely, when implemented naïvely for signals of 16,384 samples, its Block RAM (BRAM) utilization nearly exceeds the maximum available in the chosen FPGA. This utilization is a result of two factors: (1) buffers for single-precision floating-point inputs and outputs and (2) storage for twiddle factors and intermediate results. So far, we have attempted to reduce the utilization in three ways:

**S1**  **Reduced-precision arithmetic:** Our current FFT implementation uses single-precision floating-point data to ensure acceptable acquisition results, as used for Figure 8c. However, floating-point arithmetic is costly, especially in FPGAs, and may be replaced with fixed-point arithmetic with properly dimensioned formats to induce major resource savings.
**S2**  **Reduced size:** The current FFT implementation is designed for 16,384-point transforms. Yet, as mentioned above, this could be reduced to, for example, 8192-point transforms by using a lower sampling rate, leading to additional resource savings.
**S3**  **Smarter HLS:** Our current PCPS implementation makes three calls to an FFT. A crude HLS flow would synthesize these into each their own FFT module while, in reality, it may be beneficial to trade off vast savings in resource utilization for slightly reduced performance by sharing one FFT or, perhaps, at most two: one forward and one inverse. This, however, requires fine-tuning of our code using with HLS-related pragmas (e.g., allocation [38]). Additional pragmas, such as bind_storage, array_partition, and array_reshape, might also help make the FFT’s buffer utilization more efficient.

The potential effects of solutions S1 and S2 on the hardware resource budget are explored in Table 2. We provide absolute post-synthesis resource utilization estimates, in Lookup Tables (LUTs), Flip-Flops (FFs), DSP slices, and BRAMs, along with savings relative to our reference {16k, float} implementation. ap_fixed〈16,1〉 refers to a 16-bit fixed-point format with one integer bit and fifteen fractional bits. As mentioned above, BRAMs are the resource bottleneck. Combining solutions S1 and S2, we can achieve savings of almost 80% of the BRAMs using {8k, ap_fixed〈16,1〉} instead of {16k, float}. Vitis HLS also natively supports fixed-point formats with fewer than 16 bits, yet our architecture requires further adaptation to function properly with such.

It is noteworthy that post-synthesis resource utilization numbers from HLS flows tend to be associated with rather large errors [45]. This is observed for the LUT and FF numbers in our experiments too, for which the estimates can be as much as eight times off the accurate post-implementation results. However, importantly, the BRAM estimates are not affected by these errors as they result from fully specified array declarations in the C/C++ code. Additionally, we experienced that Vitis HLS produces invalid HDL code for its FFT IP when using its array-based interface as specified in [38]. We have reported this on the Xilinx forums; see  https://shorturl.at/ntvGM accessed on 14 December 2023. Initial experiments following S3 with manual allocation of nearly all non-FFT-related buffers to the UltraRAM slices available on UltraScale devices indicate that at least one full channel with acquisition and tracking can be fit on the KV260’s FPGA.

Therefore, although the current implementation requires a large amount of resources, these can be greatly reduced, and we foresee exploring the effects of the three solutions to its optimization, including the unexplored solution S3, further in our future development.

### 5.2. Results of the Tracking Stage

As the tracking stage required fewer changes compared to the acquisition stage, we successfully implemented it on the FPGA board, following the revised design presented in Section 3.2. Since this conversion did not involve any significant changes in the algorithm design (i.e., the same processing functions and data types), the tracking results are exactly the same between the Python, C/C++, and FPGA implementations. Consequently, we do not present these results here but invite the interested reader to consider our previous works for more details [9,22]. Instead, we evaluate our hardware design using the following KPIs: resource utilization, latency, and power/energy consumption estimates.

We first analyze the hardware utilization of our tracking-only Hard SyDR design, listed in Table 3. The design is implemented for a clock frequency of 100 MHz. The utilization numbers include a simple channel manager in charge of maintaining channel states. To examine the results in detail, we break down the numbers to highlight the differences in costs of the involved functions. Notably, with respect to the total available resources on the FPGA, the design consumes only 32.6% of the LUTs, 9.36% of the FFs, 23.5% of the DSPs, and 21.2% of the BRAMs. In other words, despite the FPGA on the KV260 board being an entry-level, hence “small”, Zynq device with limited resources, it easily accommodates our non-optimized design.

From Table 3, we see that the tracking function consumes most of the design’s resources. Internally, it relies heavily on double-precision floating-point arithmetic that can be costly to implement in FPGAs as certain operations, such as division and square root, are not mapped to the fabric’s DSP slices. Moreover, we notice that the mathematical functions—inverse tangent, sine, and cosine—also are costly in resources. These observations are as expected and highlight the need for a design space exploration around the data types used in tracking algorithms meant for hardware implementation.

Next, we investigate the latency of key functions in the design, including data transfers, and provide a breakdown in Table 4. The profiling results show that computing dominates the execution time, indicating a high computation-to-communication ratio and a good use of memory to minimize data movement internally to the FPGA. Note that the data transfer time does not include reading the RF data file. This constitutes a significant part of the overall processing time, but as it is a byproduct of the PYNQ flow, it is irrelevant to our hardware-focused analysis. The computations of the tracking stage dominate the remaining time.

**Figure 9:**
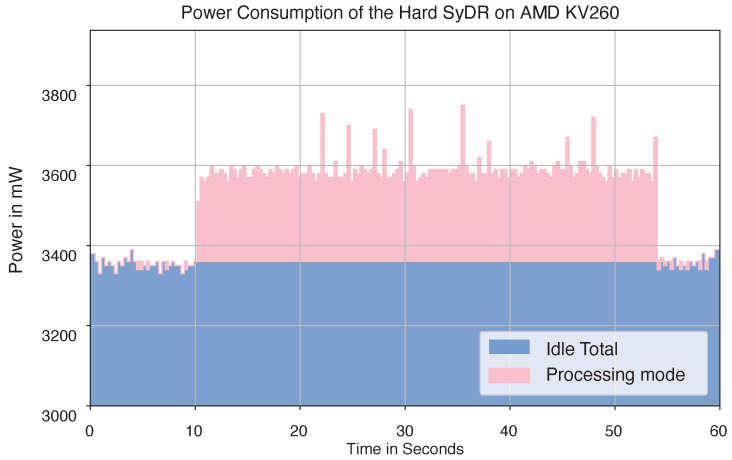
Power consumption of the Hard SyDR at idle and execution.

Finally, it is possible to measure the power and energy consumption of the complete Zynq chip using the power measurement utility built into the PYNQ flow. While the measurements therefrom include both the PS and the PL, it is possible to isolate the dynamic power consumption of the PL by running two power measurements: first, when the PL is idle, and second, when the PL implements a design and is processing data. While changes in the dynamic power consumption of the PS may disturb these measurements, we assume that the workloads the PS executes during both measurement processes are sufficiently similar to render these disturbances negligible compared with the dynamic power consumption of the PL. Table 5 provides the power measurements and energy consumption estimates based on 1000 runs of the tracking stage. The energy consumption is computed by multiplying the power numbers by the total processing time. Figure 9 provides an overview of the power consumption over time in both idle and processing modes.

## 6. Discussion and Open Challenges

In this section, we summarize the results and contributions C2–C4 listed in Section 1.

### 6.1. From Software to Hardware

In this work, we have explored how GNSS algorithms developed in a high-level programming language (Python) can be ported to hardware with a limited effort (C2). Our conversion process required partial re-development in an intermediate language (C/C++) to use HLS to produce HDL descriptions. We have not reviewed libraries to convert Python code into C/C++ code, such as Cython, for two reasons: (1) successful application of the HLS synthesis flow requires knowledge of the C code and (2) retaining a certain level of code quality from the beginning might not be possible with such tools. Moreover, one of our reasons for writing SyDR in a high-level language was to simplify the validation of any processing stages that would later be converted into lower-level languages. In particular, we use unit tests for each algorithm whose expected values are generated in Python.

Naïvely using HLS generally shortens the development time and requires less domain-specific knowledge of hardware design than using any traditional HDL would. The latter point renders it particularly interesting to GNSS algorithm researchers, who often come from a software development background. Despite some initial hiccups in our conversion due mostly to inexperience, we still believe that using HLS rather than handwriting HDL has accelerated the progress of Hard SyDR (C3).

Nevertheless, we acknowledge that using HLS is non-trivial. As mentioned before, it has well-known overheads [41,45], which require understanding of hardware design and the targeted FPGA architecture to mitigate. As shown in Section 5.1, for example, fine-tuning details of a design can reduce resource utilization or improve performance significantly. In line with this, we have a version of the Hard SyDR code that is fully synthesizable but consumes far more BRAMs than are available in the KV260’s FPGA [35].

Following this work, we plan to explore the efficiency of the HLS flow by developing HDL versions of key GNSS algorithms and, in parallel, optimizing our current design better. Moreover, we will consider the feasibility of using power consumption estimates from post-implementation simulations instead of on-board measurements. If deemed sufficiently accurate, this would allow for a truly portable algorithm evaluation flow without physical hardware.

### 6.2. Opportunities Provided by PYNQ

While the HLS development proved to be more time-consuming than expected, using the PYNQ flow was surprisingly quick (C3). Once the hardware layout is available, its integration into a Jupyter Notebook is straightforward. The PYNQ flow enabled us to rapidly port only a small portion of the SyDR code (e.g., the algorithms of interest) to an FPGA and to test it on a physical board (C4). As a result thereof, we could skip manually implementing, for example, a component to interact with a pre-recorded RF data file.

The ease of integration with PYNQ has led us to consider using multiple designs, each implementing either acquisition or tracking algorithms, rather than one complete design as an alternative strategy to meet the resource limitations of the KV260 board [35]. However, we are yet to assess the associated overheads of reconfiguring the FPGA, possibly using its partial reconfiguration features.

### 6.3. Perspectives of Using AxC

Our prime objective in this research is to enable a design space exploration of using hardware-level AxC techniques in GNSS algorithms to provide more realistic power/energy estimates than from software. However, given the challenges encountered during our work with HLS, especially regarding the FFT, we acknowledge that integrating techniques, like approximate arithmetic [10,11], is non-trivial and requires an analysis of the affected algorithms or processing stages to identify their error-resilient elements [48]. We expect to pursue such an analysis, beginning with software models of simple approximate adders and multipliers.

## 7. Conclusions

In this paper, we looked into mixing software and hardware developments within one framework, targeting benchmarking of GNSS algorithms, and accessing new comparison KPIs for energy efficiency assessment. We have explored the feasibility of porting parts of an GNSS-SDR developed in a high-level programming language into hardware while minimizing the need for re-development. Using an AMD Zynq device and the associated PYNQ flow, we provided a proof-of-concept architecture to perform benchmarking of GNSS algorithms on an FPGA board for a more realistic estimation of power/energy consumption. We first reviewed our SDR named SyDR, developed in Python, and how it can be used to obtain a first estimate of the time required by each stage of the GNSS processing chain. Consequently, we highlighted the need for more realistic power/energy estimations to improve its benchmarking capacity. In response, we presented our Hard SyDR concept that couples Python with algorithms running in an FPGA. We outlined our process of converting our algorithms in Python via C/C++ and HLS into functional hardware designs. Next, we presented the first results of our implementation, illustrating the challenges encountered. These results show a clear need for design optimization to implement a complete replica of a GNSS receiver inside the FPGA selected. Yet, the new KPIs made available by Hard SyDR can be exploited to refine our implementations further. Future work will focus on going deeper in the exploration of the HLS process and the implementation of AxC techniques to achieve significant reductions in energy consumption in next-generation GNSS algorithms.

## Figures and Tables

**Table 1:** Simulation parameters used in SyDR and Hard SyDR.

Reference Dataset		
Date		2021.11.30, ∼ 8:40 (UTC)
Duration		15 s (15,000 ms)
Location		TAU Rooftop (open-sky)
Dynamic		Static
Antenna		Novatel GPS-703-GGG
Instruments	RF Logger	NI USRP-2953R
	Clock	Spectracom GSG-6
Frequency	Center	1575.42 MHz (L1)
	Bandwidth	120 MHz
	Sampling frequency	10 MHz
	Quantization	8 bits
	I/Q	Complex
**Acquisition**		
Method		Parallel Code Phase Search
Doppler Range		± 5000 Hz, 250 Hz steps
Integration		No integration (1 ms only)
Threshold		Ratio two highest peak, 1.5
**Tracking**		
Method		Early Prompt Late
Correlator spacing		−0.5/0/0.5
DLL	PDI	0.001
	Damping ratio	0.7
	Noise bandwidth	2.0 Hz
	Loop gain	1.0
PLL	PDI	0.001
	Damping ratio	0.7
	Noise bandwidth	25.0 Hz
	Loop gain	0.25

**Table 2:** Post-synthesis resource utilization estimates and parenthesized relative savings of different configurations of the Vitis HLS FFT IP block.

FFT Size	Datatype	LUTs		FFs		DSPs		BRAMs	
**16k**	float	20,838	—	24,898	—	0	—	116.5	—
	ap_fixed〈16,1〉	19,141	(−8.14%)	23,011	(−7.58%)	0	(0%)	61	(−47.6%)
**8k**	float	14,780	(−29.1%)	15,822	(−36.5%)	0	(0%)	49	(−57.9%)
	ap_fixed〈16,1〉	13,780	(−33.9%)	14706	(−40.9%)	0	(0%)	25	(−78.5%)

**Table 3:** Post-implementation resource utilization of one tracking channel compared to the total resources available in the XCK26-C device used in the KV260 development board [35]. Percentages are relative to the total available resources of each type.

Resource	LUTs		FFs		DSPs		BRAMs	
**Available**	117,120	—	234,240	—	1248	—	144	—
**Hard SyDR**	37,552	32.1%	21,923	9.36%	293	23.5%	30.5	21.2%
Tracking	31,827	27.2%	17,201	7.34%	293	23.5%	14.5	10.1%
Early-Prompt-Late	10,587	9.04%	4254	1.82%	198	15.9%	1	0.69%
double Multiplication	245	0.21%	475	0.20%	16	1.28%	0	0.00%
double Cosine/Sine	8087	6.90%	2349	1.00%	182	14.6%	0	0.00%
Others	2255	1.93%	1430	0.61%	0	0.00%	1	0.69%
double Add. and Sub.	2891	2.47%	852	0.36%	6	0.00%	0	0.00%
double Multiplication	612	0.52%	561	0.24%	16	1.28%	0	0.00%
double Division	6999	5.98%	4370	1.87%	0	0.00%	0	0.00%
double Square Root	3432	2.93%	2428	1.04%	0	0.00%	0	0.00%
double Power	2948	2.52%	1353	0.58%	73	10.5%	10.5	7.29%
double Modulo	766	0.65%	336	0.14%	0	0.00%	0	0.00%
double Inverse Tangent	2548	2.18%	606	0.26%	0	0.00%	2	1.39%
Others	1044	0.89%	2441	1.04%	0	0.00%	1	0.69%
Channel Retrieve	2324	1.98%	514	0.22%	0	0.00%	0	0.00%
Channel Store	1542	1.32%	514	0.22%	0	0.00%	0	0.00%
Channel Init	1276	1.09%	513	0.22%	0	0.00%	0	0.00%
Others	583	0.50%	3181	1.36%	0	0.00%	16	11.1%

**Table 4:** Task execution time of the Hard SyDR design on the FPGA.

Tasks	Time	Ratio
Reading of Inputs	994 μs	2.25%
Retrieve Channel States	5.1 μs	0.01%
Tracking	43,100 μs	97.7%
Preserve Channel States	5.1 μs	0.01%
Total	44,104 μs	100%

**Table 5:** Power consumption of the design and the energy consumption of FPGA.

Item	Time (s)	Mean Power (mW)	STD Power (mW)	Mean Energy (mJ)
System Idle	—	3360.1	45.3	—
FPGA	44.3	236.8	48.3	10,490

**Figure A1:**
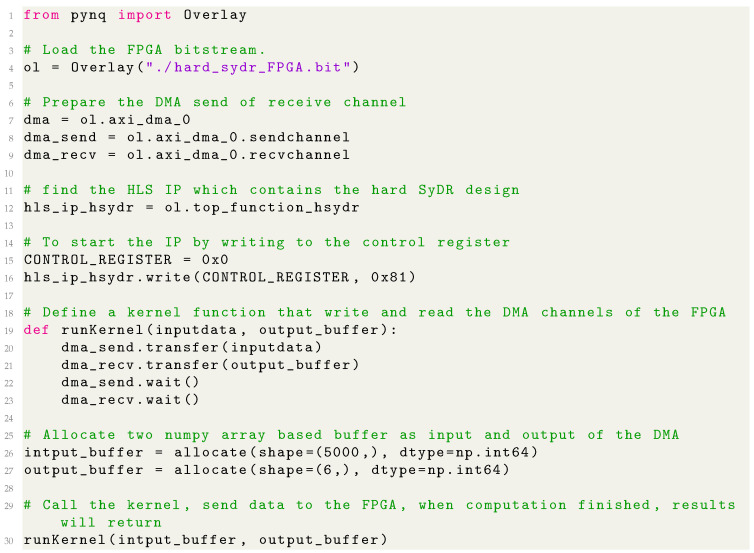
Python code for FPGA interaction.

## Data Availability

Data are contained within the article.

## Abbreviations

ADC	Analog-to-Digital Converter.
ASIC	Application-Specific Integrated Circuit.
AxC	Approximate Computing.
AXI	Advanced eXtensible Interface.
BRAM	Block RAM.
CPU	Central Processing Unit.
DGNSS	Differential GNSS.
DMA	Direct Memory Access.
DSP	Digital Signal Processing.
FFT	Fast Fourier Transform.
FPGA	Field-Programmable Gate Array.
GNSS	Global Navigation Satellite System.
HDL	Hardware Description Language.
HLS	High-Level Synthesis.
IC	Integrated Circuit.
IP	Internet Protocol.
KPI	Key Performance Indicator.
LUT	Lookup Table.
ML	Machine Learning.
NoC	Network on Chip.
PL	Programmable Logic.
PPP	Precise Point Positioning.
PRN	Pseudo-Random Noise.
PS	Processing System.
PU	Processing Unit.
RAM	Random Access Memory.
RF	Radio Frequency.
SDR	Software-Defined Radio.
SNR	Signal to Noise Ratio.
SPP	Standard Point Positioning.
TOW	Time of Week.
USRP	Universal Software Radio Peripheral.
VHDL	Very-high Speed Integrated Circuit Hardware Description Language

## Appendix A

Snippet of Python code illustrating how to use PYNQ to interact with a design in the FPGA on the Zynq platform. The example code programs the FPGA and enables the design before transferring an array of 5000 64-bit integers to it and receiving six 64-bit integers in return. Our Jupyter notebook used for initial experiments with Hard SyDR extends upon this code by looping over the runKernel function till a pre-determined number of milliseconds of RF data have been processed.
